# Supplementary material for: Vitamin D3 Supplementation at 5000 IU Daily for the Prevention of Influenza-like Illness in Healthcare Workers: A Pragmatic Randomized Clinical Trial
Source: Nutrients. 2022 Dec 30;15(1):180. doi: 10.3390/nu15010180 (PMC9823308; doi:10.3390/nu15010180)
Supplement: Supplementary file 1 [file nutrients-15-00180-s001.zip › Supplement 2.pdf]

Supplement 2. Adverse events (AE) and relatedness among intervention group during 9-month study period

Adverse events (AE) and relatedness among intervention group during 9-month study period				
**Adverse Events = 388**	**AE (%)**	Probably Related	Possibly Related	Unrelated
Fatigue	**36 (9.3%)**	0	4	32
Urinary frequency	**24 (6.2%)**	1	7	16
Nausea	**23 (5.9%)**	0	8	15
Lower back pain	**21 (5.4%)**	0	2	19
Thirst/dry mouth	**17 (4.4%)**	1	5	11
Abdominal pain	**11 (2.8%)**	1	4	6
Back pain	**11 (2.8%)**	0	1	10
Palpitations	**10 (2.6%)**	0	5	5
Stomach upset	**10 (2.6%)**	0	4	6
Urinary tract infection	**9 (2.3%)**	0	1	8
Headache	**8 (2.1%)**	0	0	8
GERD	**7 (1.8%)**	0	1	6
Depression	**6 (1.5%)**	0	1	5
Lack of energy	**6 (1.5%)**	0	1	5
Anxiety	**5 (1.3%)**	0	0	5
Bone pain	**5 (1.3%)**	0	0	5
Constipation	**5 (1.3%)**	0	4	1
High blood pressure	**5 (1.3%)**	0	1	4
Muscle weakness	**5 (1.3%)**	0	0	5
Anxiety/depression	**4 (1.0%)**	0	0	4
Sinus infection/sinusitis	**4 (1.0%)**	0	0	4
Joint pain	**3 (0.8%)**	0	0	3
Rash	**3 (0.8%)**	0	1	2
Stomach upset/nausea	**3 (0.8%)**	0	1	2
Stomach virus	**3 (0.8%)**	0	0	3
Strong heartbeat	**3 (0.8%)**	0	2	1
Vomiting	**3 (0.8%)**	0	1	2
Bronchitis	**2 (0.5%)**	0	0	2
Dog bite	**2 (0.5%)**	0	0	2
Hives	**2 (0.5%)**	0	1	1
Lightheadedness	**2 (0.5%)**	0	0	2
Sciatica	**2 (0.5%)**	0	0	2
Tooth infection	**2 (0.5%)**	0	0	2
Upper back pain	**2 (0.5%)**	0	1	1
Weight gain	**2 (0.5%)**	0	0	2
Abdominal cramping/loose stools	**1 (0.3%)**	0	1	0
Abdominal muscle pain	**1 (0.3%)**	0	0	1
Abdominal/bloating	**1 (0.3%)**	0	0	1
Anemia	**1 (0.3%)**	0	0	1
Angioedema	**1 (0.3%)**	0	0	1

Adverse events (AE) and relatedness among intervention group during 9-month study period				
**Adverse Events = 388**	**AE (%)**	Probably Related	Possibly Related	Unrelated
Attention deficit	**1 (0.3%)**	0	0	1
Bad taste in mouth	**1 (0.3%)**	0	0	1
Bloating, sense of fullness	**1 (0.3%)**	0	0	1
Body aches, joint aches, pain in legs and knees	**1 (0.3%)**	0	0	1
Bone and joint pain	**1 (0.3%)**	0	1	0
Bone pain-mild joint aches and mild post exercise joint discomfort.	**1 (0.3%)**	0	0	1
Bright red blood per rectum	**1 (0.3%)**	0	0	1
Burning sensation arms and legs	**1 (0.3%)**	1	0	0
Cellulitis	**1 (0.3%)**	0	0	1
Chest Pain and palpitations	**1 (0.3%)**	0	1	0
Confusion	**1 (0.3%)**	0	0	1
Conjunctivitis of the left eye	**1 (0.3%)**	0	0	1
Consistent severe itching of right inner ear	**1 (0.3%)**	0	0	1
Cramping and soreness in both legs	**1 (0.3%)**	0	1	0
Degenerative disc disease	**1 (0.3%)**	0	0	1
Diabetes type II	**1 (0.3%)**	0	0	1
Diarrhea	**1 (0.3%)**	0	0	1
Dizziness	**1 (0.3%)**	0	0	1
Fascia tightness/stiffness-neck, shoulders, back, arms, legs, feet	**1 (0.3%)**	0	0	1
Feeling hot	**1 (0.3%)**	0	0	1
Fibromyalgia	**1 (0.3%)**	0	0	1
Flank pain	**1 (0.3%)**	0	0	1
Fluid retention	**1 (0.3%)**	0	0	1
Food poisoning	**1 (0.3%)**	0	0	1
Foot injury from running	**1 (0.3%)**	0	0	1
Gallbladder polyps	**1 (0.3%)**	0	0	1
Ganglion cyst 3rd MCP and index finger right hand	**1 (0.3%)**	0	0	1
Gastroparesis	**1 (0.3%)**	0	0	1
GI upset	**1 (0.3%)**	0	1	0
GI virus	**1 (0.3%)**	0	0	1
Gum infection	**1 (0.3%)**	0	0	1
H. pylori	**1 (0.3%)**	0	0	1
Hair loss	**1 (0.3%)**	0	0	1
Hand pain	**1 (0.3%)**	0	0	1
Having a bowel movement more often	**1 (0.3%)**	0	1	0
Heaviness in lower extremities	**1 (0.3%)**	0	0	1
Hematemesis	**1 (0.3%)**	0	0	1
High heartbeat	**1 (0.3%)**	0	0	1
Hypersensitivity to sun	**1 (0.3%)**	1	0	0
Increase in occurrence of pain in right hip and right knee	**1 (0.3%)**	0	0	1
Increased drinking and urination	**1 (0.3%)**	0	1	0

Adverse events (AE) and relatedness among intervention group during 9-month study period				
**Adverse Events = 388**	**AE (%)**	Probably Related	Possibly Related	Unrelated
Increased frequency of IBS pain	**1 (0.3%)**	0	0	1
Infected ingrown toenail	**1 (0.3%)**	0	0	1
Infected R toe	**1 (0.3%)**	0	0	1
Insomnia-intermittent	**1 (0.3%)**	0	0	1
Jaw pain	**1 (0.3%)**	0	1	0
Kidney stone	**1 (0.3%)**	0	0	1
Knee pain	**1 (0.3%)**	0	0	1
Knee pain and shin pain	**1 (0.3%)**	0	1	0
Labral tear in hip joint	**1 (0.3%)**	0	0	1
Left knee pain	**1 (0.3%)**	0	0	1
Low B12	**1 (0.3%)**	0	0	1
Low back pain-intermittent	**1 (0.3%)**	0	0	1
Low iron	**1 (0.3%)**	0	0	1
Memory issue	**1 (0.3%)**	0	0	1
Menstrual issue	**1 (0.3%)**	0	0	1
More frequent urination and urge	**1 (0.3%)**	0	0	1
Mouth lesions	**1 (0.3%)**	1	0	0
Muscle pain	**1 (0.3%)**	0	0	1
Muscle pain in arms	**1 (0.3%)**	0	0	1
Muscle spasms for 2-3 days, both sides of rib cage	**1 (0.3%)**	0	0	1
Neck pain	**1 (0.3%)**	0	0	1
Nodules on right lower forearm and right foot near ankle	**1 (0.3%)**	0	0	1
Nodules on throat	**1 (0.3%)**	0	0	1
Occasional stomach pains	**1 (0.3%)**	0	0	1
Oral tenderness	**1 (0.3%)**	1	0	0
Otitis externa	**1 (0.3%)**	0	0	1
Pain in fingers-possibly arthritis	**1 (0.3%)**	0	0	1
Pain-knee	**1 (0.3%)**	0	0	1
Pelvic pain	**1 (0.3%)**	0	0	1
Pulled muscle left elbow	**1 (0.3%)**	0	0	1
PVCs	**1 (0.3%)**	0	0	1
Rapid heartbeat	**1 (0.3%)**	0	1	0
Raynaud's	**1 (0.3%)**	0	0	1
Recurrent strep throat infections	**1 (0.3%)**	0	0	1
Rib pain	**1 (0.3%)**	0	1	0
Ridges in nails	**1 (0.3%)**	0	0	1
Right ankle, left knee and left wrist worsened pain	**1 (0.3%)**	0	0	1
Right big toe swollen/stiff	**1 (0.3%)**	0	0	1
Right knee pain	**1 (0.3%)**	0	0	1
Right leg pain	**1 (0.3%)**	0	0	1
Right ovarian cyst	**1 (0.3%)**	0	0	1
Right shoulder pain	**1 (0.3%)**	0	0	1

**Adverse events (AE) and relatedness among intervention group during 9-month study period**				
**Adverse Events = 388**	**AE (%)**	Probably Related	Possibly Related	Unrelated
Right shoulder stiffening	**1 (0.3%)**	0	0	1
Ruptured Meckel's diverticulum	**1 (0.3%)**	0	0	1
Scabies	**1 (0.3%)**	0	0	1
Shingles	**1 (0.3%)**	0	0	1
Short moments of forgetting things	**1 (0.3%)**	0	0	1
Shoulder pain-frozen shoulder	**1 (0.3%)**	0	0	1
Shoulder surgery	**1 (0.3%)**	0	0	1
Spasms in shoulder resulting from surgery	**1 (0.3%)**	0	0	1
Stomach cramping	**1 (0.3%)**	0	0	1
Stomach pain	**1 (0.3%)**	0	0	1
Stomach pain intermittent	**1 (0.3%)**	0	1	0
Stomach upset and constipation - intermittent	**1 (0.3%)**	0	0	1
Stye in left eye	**1 (0.3%)**	0	0	1
Swelling of feet, weakness in hands, discomfort in hands and feet	**1 (0.3%)**	0	0	1
Swollen gland near jaw	**1 (0.3%)**	0	0	1
Swollen gum	**1 (0.3%)**	0	0	1
Tachycardia and chest pain thinks it was indigestion	**1 (0.3%)**	0	1	0
Tachycardia and pressure in chest	**1 (0.3%)**	0	0	1
Thyroid antibodies elevated	**1 (0.3%)**	0	0	1
Thyroidectomy	**1 (0.3%)**	0	0	1
Tick bite	**1 (0.3%)**	0	0	1
TMJ	**1 (0.3%)**	0	1	0
Toes, and fingers twitching, muscle twitching	**1 (0.3%)**	0	0	1
Unable to catch breath	**1 (0.3%)**	0	0	1
Unknown allergic reaction	**1 (0.3%)**	0	0	1
Upper back pain and Upper leg pain	**1 (0.3%)**	0	0	1
Urinary urgency	**1 (0.3%)**	0	0	1
Vaginal burning	**1 (0.3%)**	0	0	1
Vertigo	**1 (0.3%)**	0	0	1
Worsened acne	**1 (0.3%)**	0	1	0
Worsened neck, shoulder, back stiffness and soreness	**1 (0.3%)**	0	0	1
Worsening back and right leg pain	**1 (0.3%)**	1	0	0
Wound infection	**1 (0.3%)**	0	0	1
Yeast infections	**1 (0.3%)**	0	0	1
**Total Adverse Events (AEs)**	**100%**	**8**	**71**	**309**
